# Green synthesis of silk sericin-capped silver nanoparticles and their potent anti-bacterial activity

**DOI:** 10.1186/1556-276X-9-79

**Published:** 2014-02-17

**Authors:** Pornanong Aramwit, Nipaporn Bang, Juthamas Ratanavaraporn, Sanong Ekgasit

**Affiliations:** 1 Bioactive Resources for Innovative Clinical Applications Research Unit and Department of Pharmacy Practice, Faculty of Pharmaceutical Sciences, Chulalongkorn University, 254 PhyaThai Road, Patumwan, Bangkok 10330, Thailand; 2 Department of Chemical Engineering, Faculty of Engineering, Chulalongkorn University, 254 PhyaThai Road, Patumwan, Bangkok 10330, Thailand; 3 Sensor Research Unit, Department of Chemistry, Faculty of Science, Chulalongkorn University, 254 PhyaThai Road, Patumwan, Bangkok 10330, Thailand

**Keywords:** Green synthesis, Silver nanoparticle, Silk sericin, Alkaline degradation, Anti-bacterial activity

## Abstract

In this study, a ‘green chemistry’ approach was introduced to synthesize silk sericin (SS)-capped silver nanoparticles (AgNPs) under an alkaline condition (pH 11) using SS as a reducing and stabilizing agent instead of toxic chemicals. The SS-capped AgNPs were successfully synthesized at various concentrations of SS and AgNO_3_, but the yields were different. A higher yield of SS-capped AgNPs was obtained when the concentrations of SS and AgNO_3_ were increased. The SS-capped AgNPs showed a round shape and uniform size with diameter at around 48 to 117 nm. The Fourier transform infrared (FT-IR) spectroscopy result proved that the carboxylate groups obtained from alkaline degradation of SS would be a reducing agent for the generation of AgNPs while COO^−^ and NH_2_ ^+^ groups stabilized the AgNPs and prevented their precipitation or aggregation. Furthermore, the SS-capped AgNPs showed potent anti-bacterial activity against various gram-positive bacteria (minimal inhibitory concentration (MIC) 0.008 mM) and gram-negative bacteria (MIC ranging from 0.001 to 0.004 mM). Therefore, the SS-capped AgNPs would be a safe candidate for anti-bacterial applications.

## Background

Over the last decades, silver nanoparticles (AgNPs) have been widely used in catalytic, optic, electronic, and other applications due to their unique size-dependent properties and high surface-to-volume ratio, which are significantly different from those of the corresponding bulk materials [1]. Recently, there has been a great deal of interest in the effective anti-bacterial/anti-fungal activity of AgNPs [2-5]. In fact, it is well known that Ag ions (Ag^+^) and Ag-based compounds have strong biocidal effects on as many as 12 species of bacteria including *Escherichia coli*[6]. Das et al. showed that AgNPs with a 12-nm size could be used as effective growth inhibitors against *Bacillus subtilis*, *Staphylococcus aureus*, and *Pseudomonas aeruginosa*[3]. Kim et al. reported that yeast and *E. coli* were inhibited at the low concentration of AgNPs [7]. Furthermore, silver exhibits low toxicity and minimal risk in the human body [8].

AgNPs can be synthesized by a variety of methods such as reverse micelle process [9], chemical reduction [10], microwave dielectric heating reduction [11], ultrasonic irradiation [12], radiolysis [13], solvothermal synthesis [14], electrochemical synthesis [15], bacterial synthesis [16], etc. Among these methods, chemical reduction is one of the easiest and widely used techniques. Solomon et al. have reported the chemical reduction of silver nitrate using sodium borohydride to synthesize stable and non-aggregated AgNPs [17]. Sodium dodecyl sulfate, sodium citrate, and hydrazine hydrate solution were also used as stabilizing and reducing agents to prepare AgNPs with high anti-microbial activity against gram-positive bacteria [18]. However, these chemical methods use organic solvents and toxic reducing agents, consume high energy, and require difficult waste treatment. Recently, researchers have an increasing awareness about the environment. The use of toxic chemicals and solvents should be avoided, contributing to the emergence of ‘green chemistry’ for the synthesis of AgNPs [19-23]. Utilizations of environmentally friendly or naturally derived materials are some of the key issues of a green synthesis strategy [19-23]. Various types of microorganisms have been reported to synthesize AgNPs either intra- or extracellularly [19,20]. Also, stable AgNPs could be synthesized by using polysaccharides such as starch as both reducing and stabilizing agents [21,22]. AgNPs were synthesized by autoclaving a solution of AgNO_3_ and starch [21]. Starch undergoes hydrothermal hydrolysis in an autoclave to produce glucose. Thus, starch can be used instead of pure glucose for the synthesis of AgNPs. In addition to polysaccharides, proteins, which are naturally abundant non-toxic materials and available from various sources, are introduced for AgNP synthesis. Zhao et al. have synthesized a AgNP-embedded soy protein isolation (SPI) film [24]. The whole reaction process was carried out by exposure to white light at ambient temperature, which is highly energy-efficient and eco-friendly. Moreover, the AgNP-embedded SPI film showed an effective anti-microbial activity against both gram-positive and gram-negative bacteria. Sasikala et al. have introduced the capabilities of the miracle bean soybean *Glycine max* as a stabilizer in the production of AgNPs [25]. Irwin et al. reported that keratin-stabilized AgNPs at 0.3 to 3 μM completely inhibited the growth of an equivalent volume of *ca.* 10^3^ to 10^4^ colony-forming units per milliliter (CFU/mL) of *S. aureus*, *Salmonella typhimurium*, or *E. coli*[26].

In this study, silk sericin protein was introduced for AgNP synthesis. Silk sericin (SS) is a water-soluble protein extracted from silkworms. Currently, SS is considered as a waste product from the textile industry. It is highly hydrophilic with strong polar side chains such as hydroxyl, carboxyl, and amino groups. Recently, SS has been widely used in biomaterial applications due to its biocompatibility, biodegradability, and anti-oxidative and bioactive activities. We herein introduced SS as a reducing and stabilizing agent for AgNP synthesis. Due to the results, SS can be used instead of other natural products to easily produce AgNPs. The effects of reaction conditions including the pH value and concentrations of SS and silver nitrate (AgNO_3_) solutions on AgNP formation were investigated via a UV-visible (UV-Vis) spectrophotometer, transmission electron microscope (TEM), and colorimeter. The size and zeta potential of the SS-capped AgNPs were determined by using Zetasizer. The chemical structure of the SS-capped AgNPs was analyzed by attenuated total reflection Fourier transform infrared (ATR FT-IR) spectroscopy. The anti-microbial activity of the SS-capped AgNPs against gram-positive and gram-negative bacteria was evaluated.

## Methods

### Materials

Fresh bivoltine white-shell cocoons of *Bombyx mori* produced in a controlled environment were kindly supplied by Chul Thai Silk Co., Ltd. (Petchaboon province, Thailand). Silver nitrate (AgNO_3_), sodium hydroxide (NaOH), and other chemicals were of analytical grade and used without further purification.

### Preparation of silk sericin solution

The silkworm cocoons were cut into small pieces, and SS was extracted using a high-temperature and high-pressure degumming technique [27]. Briefly, the cocoons were put into deionized (DI) water and then autoclaved at 120°C for 60 min. After filtration through a filter paper to remove fibroin fibers, the SS solution was concentrated until the desired concentration was achieved (approximately 7 wt%, measured by the BCA protein assay kit, Pierce, Rockford, IL, USA). This SS solution was used as a stock solution. The molecular weight of the SS obtained ranged from 25 to 150 kDa, as reported previously [28].

### Synthesis of SS-capped AgNPs

The SS solution was diluted to 5, 10, and 20 mg/mL, and NaOH was added to adjust the pH of the SS solution to be 9 and 11. The prepared SS solution was added to the AgNO_3_ solution (1, 5, and 10 mM) under constant stirring. The mixture was stirred at room temperature overnight. The transparent solution which turned yellow indicated the formation of SS-capped AgNPs.

### Characterization of SS-capped AgNPs

UV-Vis absorption spectra of the SS-capped AgNPs were measured using a UV-Vis spectrophotometer (PerkinElmer LAMBDA 25, Waltham, MA, USA), from 300 to 600 nm, to evaluate the formation and yield of SS-capped AgNPs. For the stability test, the SS-capped AgNP suspension was stored at different temperatures (4°C, 25°C, and 37°C) and the yield was analyzed at each pre-determined time. The concentrations of formed AgNPs were obtained from the calibration method. To construct the calibration curve, AgNP colloid standards at various initial AgNO_3_ concentrations were prepared by reducing AgNO_3_ with NaBH_4_ in the SS solution. The amount of NaBH_4_ used in the reaction was excessive, and the dissolved silver ions completely transformed into metallic silver. The characteristic plasmon absorption at 420 nm was plotted against the initial concentration of AgNO_3_ and employed as a calibration curve. The plasmon absorption intensity at 420 nm directly related to the amount of AgNPs formed. The color of the SS-capped AgNP suspension was determined using a colorimeter (Konica Minolta CR 400, Chiyoda-ku, Japan). The CIELab scale was used; lightness (L*) and chromaticity parameter b* (yellow-blue) were measured. The size and zeta potential of SS-capped AgNPs were determined by Zetasizer Nano Range (Malvern Instruments Ltd, Malvern, UK). A drop of SS-capped AgNPs was placed on carbon-coated copper grids and observed on TEM (Hitachi H-7650, Chiyoda-ku, Japan).

The chemical structure of the SS-capped AgNPs was analyzed by ATR FT-IR spectroscopy. Briefly, the SS-capped AgNP suspension was dropped on a glass slide and left to dry overnight under an ambient condition. The ATR spectrum was collected by a germanium micro-internal reflective element (IRE) attached onto the built-in × 15 infrared objective. A sufficient contact between the tip of the IRE and the sample was achieved by raising the sample stage towards the IRE. The degree of contact was monitored by a built-in pressure sensor on the sample stage. The ATR spectra were collected via a continuum infrared microscope attached to a Nicolet 6700 FT-IR spectrometer (Thermo Fisher Scientific, Waltham, MA, USA) using a 256-scan co-addition at a resolution of 4 cm^−1^ with a built-in nitrogen-cooled mercury-cadmium-telluride (MCT) detector.

### Evaluation of anti-bacterial activities of SS-capped AgNPs

All bacterial experiments were performed in a laminar flow hood according to the full aseptic technique protocol. The researchers wore a cap, a mask, and gloves during the experiment to prevent the contamination of harmful bacteria. The anti-bacterial activities of SS-capped AgNPs were analyzed by broth dilution method against six different pathogenic microorganisms including gram-positive bacteria (*Bacillus subtilis*, *S. aureus*, and methicillin-resistant *S. aureus* (MRSA)) and gram-negative bacteria (*E. coli*, *P. aeruginosa*, and *Acinetobacter baumannii*). The pure cultures of bacteria were subcultured on Mueller-Hinton agar (MHA). Each strain was inoculated into soybean casein digest (tryptic soy broth (TSB)) for 4 to 6 h at 37°C. The growth cultures were diluted to 5 × 10^5^ CFU/mL. AgNP suspension (1 mL) was added to the mixture of TSB (1 mL) and the bacterial culture (1 mL). After incubation at 37°C for 24 h, the minimal inhibitory concentration (MIC) was examined. It is expressed as the lowest dilution which inhibited growth, judged by the lack of turbidity in the tube. After the bacterial experiment, the used cap, mask, and gloves were autoclaved before disposal. The flasks were autoclaved for sterilization, and the area was disinfected with 70% ethanol.

### Statistical analysis

All the results were statistically analyzed by the unpaired Student's *t* test, and *p* < 0.05 was considered to be statistically significant. Data were expressed as the mean ± standard deviation.

## Results and discussion

**Figure 1:**
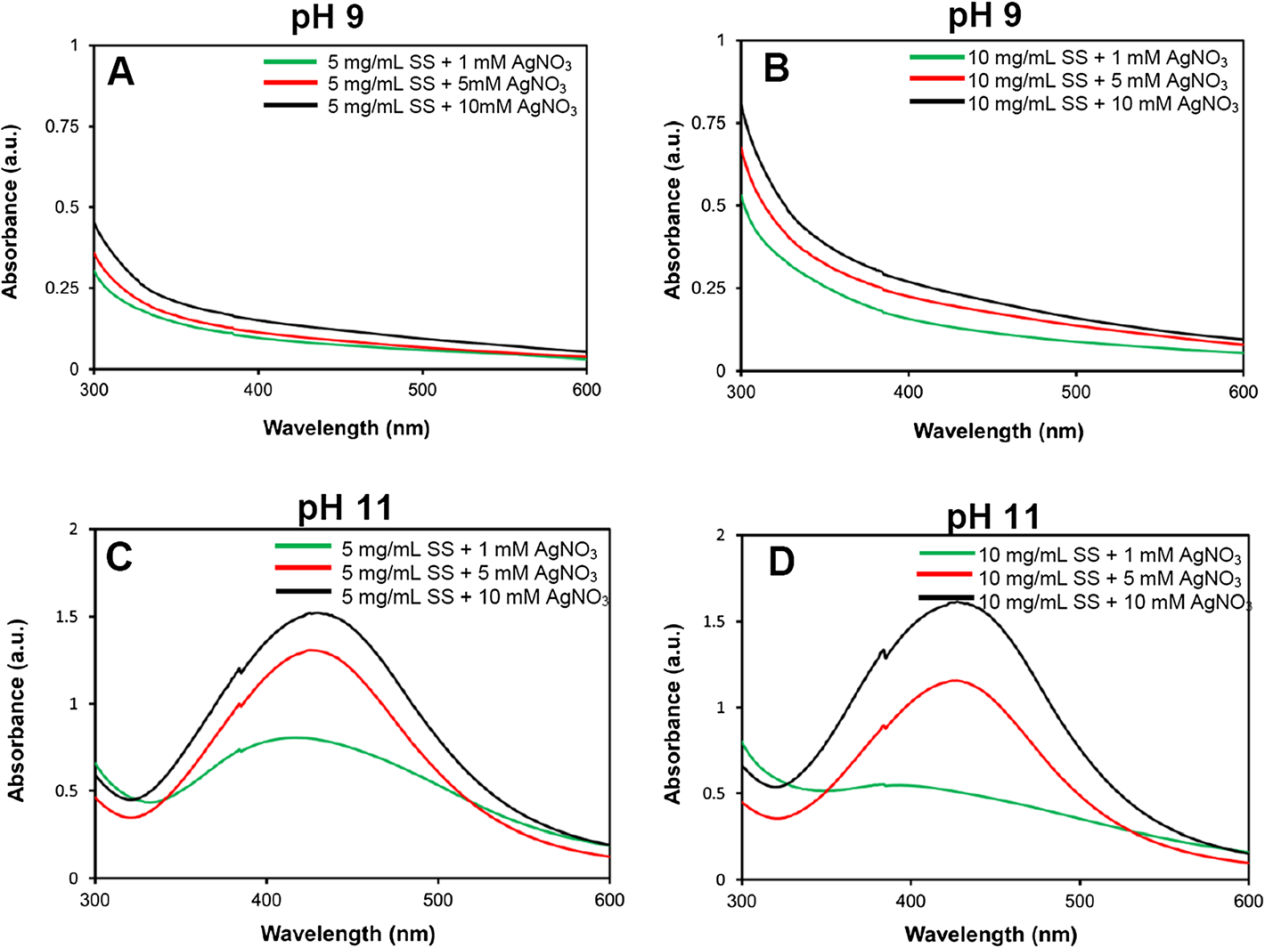
**Formation of SS-capped AgNPs at pH 9 and 11. (A)** 5 mg/mL SS + 1, 5, 10 mM AgNO_3_ at pH 9, **(B)** 10 mg/mL SS + 1, 5, 10 mM AgNO_3_ at pH 9, **(C)** 5 mg/mL SS + 1, 5, 10 mM AgNO_3_ at pH 11, **(D)** 10 mg/mL SS + 1, 5, 10 mM AgNO_3_ at pH 11.

In this work, SS was selected as a reducing and a stabilizing agent for the synthesis of AgNPs under an alkaline condition due to its being environmentally friendly, biocompatibility, and functional groups with reducing potential. The effects of pH and concentrations of AgNO_3_ and SS on the formation of SS-capped AgNPs are shown in Figure 1. It was found that the SS-capped AgNPs could not be formed at pH 9 at any concentration of SS and AgNO_3_. On the other hand, at pH 11, all concentrations of SS (5 and 10 mg/mL) and AgNO_3_ (1, 5, and 10 mM) formed SS-capped AgNPs with different yields. This is because the functional groups with reducing potential of SS could be obtained from the alkaline degradation [29-31]. SS is a hydrophilic protein with strong polar side chains such as hydroxyl, carboxyl, and amino groups. It was supposed that the SS degraded under strong alkaline condition would generate the reducing species that could reduce silver ions [21]. Furthermore, the abundant hydroxyl groups of SS were expected to complex with the silver ion and prevent aggregation or precipitation of AgNPs [21,32]. The effect of the acidic-alkaline treatment of soluble starch on the synthesis of metal nanoparticles was reported by Tongsakul et al. [29]. They found that degraded intermediates with reducing potential (i.e., aldehyde and α-hydroxy ketone) of starch are concomitantly generated when the alkaline concentration is greater than 0.025 M and the *in situ* generated species could completely reduce platinum ions (20 mM) and sufficiently stabilize the obtained platinum nanoparticles (5 mM) of uniform particle size (2 to 4 nm).

The SS-capped AgNPs had a yellow-brown color with a typical absorption peak of AgNPs at 420 nm [21]. The yield of SS-capped AgNPs increased with the increasing concentration of AgNO_3_. However, the concentration of SS seemed to show less effect on the yield of SS-capped AgNPs. Yields of SS-capped AgNPs synthesized at pH 11 are presented in Table 1. It was clearly seen that the yields of SS-capped AgNPs synthesized from 10 mg/mL SS + 1 mM AgNO_3_ were the lowest (0.04 mM) while those synthesized from 10 mg/mL SS + 10 mM AgNO_3_ were the highest (0.93 mM) according to the absorption intensity and AgNP concentration standard curve. At both 5 and 10 mg/mL SS, AgNO_3_ at 5 and 10 mM produced 0.66 to 0.93 mM SS-capped AgNPs. The colors of the AgNP suspension were quantitatively evaluated as presented in Table 2. L* and b* indicated the lightness and yellow-blue color of the suspension, respectively. The AgNP suspensions synthesized from 5 or 10 mg/mL SS + 1 mM AgNO_3_ were lighter (higher L* values) and more likely yellow (higher b* values) than those synthesized from 5 or 10 mg/mL SS + 5 mM AgNO_3_. The lighter color of the AgNP suspension indicated a lower yield of SS-capped AgNPs than the darker (brown) suspension. Thus, the higher concentration of AgNO_3_ produced the higher yield of SS-capped AgNPs.

**Table 1:** **Yield, size, and zeta potential of SS-capped AgNPs synthesized from SS and AgNO**_**3 **_**at pH 11**

**Sample**	**Yield of AgNPs (mM)**	**Size (nm)**	**Zeta potential (mV)**
5 mg/mL SS + 1 mM AgNO_3_	0.06	95.8 ± 0.9	−23.7 ± 0.6
5 mg/mL SS + 5 mM AgNO_3_	0.75	48.8 ± 0.1*	−25.2 ± 0.2
5 mg/mL SS + 10 mM AgNO_3_	0.88	55.2 ± 0.6*	−17.1 ± 0.6
10 mg/mL SS + 1 mM AgNO_3_	0.04	117.0 ± 6.8	−22.0 ± 0.1
10 mg/mL SS + 5 mM AgNO_3_	0.66	48.1 ± 0.2*	−25.5 ± 0.9
10 mg/mL SS + 10 mM AgNO_3_	0.93	63.6 ± 1.6*	−18.8 ± 2.3

*p < 0.05, significant against the values of SS-capped AgNPs synthesized from 1 mM AgNO_3_ at the corresponding SS concentration.

**Table 2:** **Color quantitative results of SS-capped AgNP suspension synthesized from SS and AgNO**_**3 **_**at pH 11**

**Sample**	L*	b*
5 mg/mL SS + 5 mM AgNO_3_	17.7 ± 0.0	0.3 ± 0.0
10 mg/mL SS + 5 mM AgNO_3_	17.7 ± 0.4	0.4 ± 0.1

L* indicates brightness ranging from black (L* = 0) to white (L* = 100). b* indicates blue-yellow color, value ranged from –60 (blue) to 60 (yellow).

**Figure 2:**
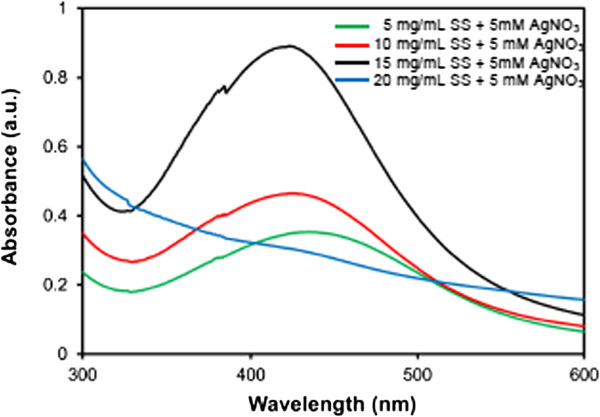
**Formation of SS-capped AgNPs synthesized from SS and AgNO**_**3 **_**at pH 11.**

The effects of the wider range of SS concentration (5, 10, 15, and 20 mg/mL) at a fixed concentration of AgNO_3_ (5 mM) on the formation of SS-capped AgNPs were further investigated, as shown in Figure 2. It was found that 5, 10, and 15 mg/mL of SS + 5 mM of AgNO_3_ could produce SS-capped AgNPs and the yield increased with the increasing concentration of SS (0.47, 0.63, and 1.26 mM, respectively), as presented in Table 3. Interestingly, at 20 mg/mL of SS, the peak absorption at 420 nm was not observed. The mechanism of this phenomenon was not clearly understood at present. It was supposed that the high SS concentration provided excessive stabilization of Ag^+^. As a result, Ag^+^ was slowly reduced. An increase in the absorption at 420 nm or intense yellow color over time was observed.

**Table 3:** **Yield of SS-capped AgNPs synthesized from SS and AgNO**_**3 **_**at pH 11**

**Sample**	**Yield of AgNPs (mM)**
5 mg/mL SS + 5 mM AgNO_3_	0.47
10 mg/mL SS + 5 mM AgNO_3_	0.63
15 mg/mL SS + 5 mM AgNO_3_	1.26
20 mg/mL SS + 5 mM AgNO_3_	-

**Figure 3:**
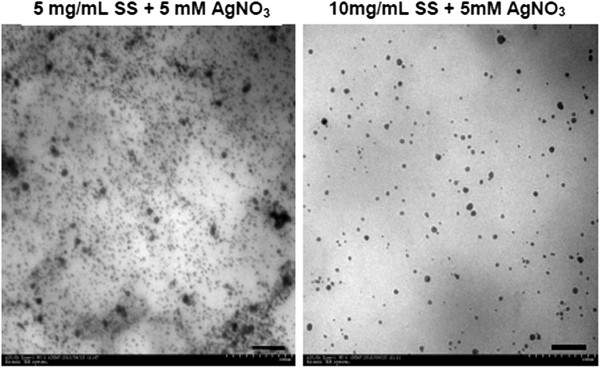
**Morphology of SS-capped AgNPs synthesized from SS and AgNO**_**3 **_**at pH 11, observed on TEM.** Scale bar = 1 μm.

Sizes and zeta potentials of SS-capped AgNPs synthesized at pH 11 are presented in Table 1. The SS-capped AgNPs synthesized from 5 or 10 mg/mL SS + 1 mM AgNO_3_ showed the largest size (95.8 and 117 nm, respectively). When the concentration of AgNO_3_ was increased to 5 or 10 mM, SS-capped AgNPs with smaller size (48 to 63 nm) were formed. However, it seemed that sizes of all nanoparticles were in a similar range (48 to 117 nm). TEM images in Figure 3 qualitatively show the round shape of SS-capped AgNPs and confirm their size as reported in Table 1. Zeta potentials of all SS-capped AgNPs were around −25 to −17 mV, indicating their high stability.

**Figure 4:**
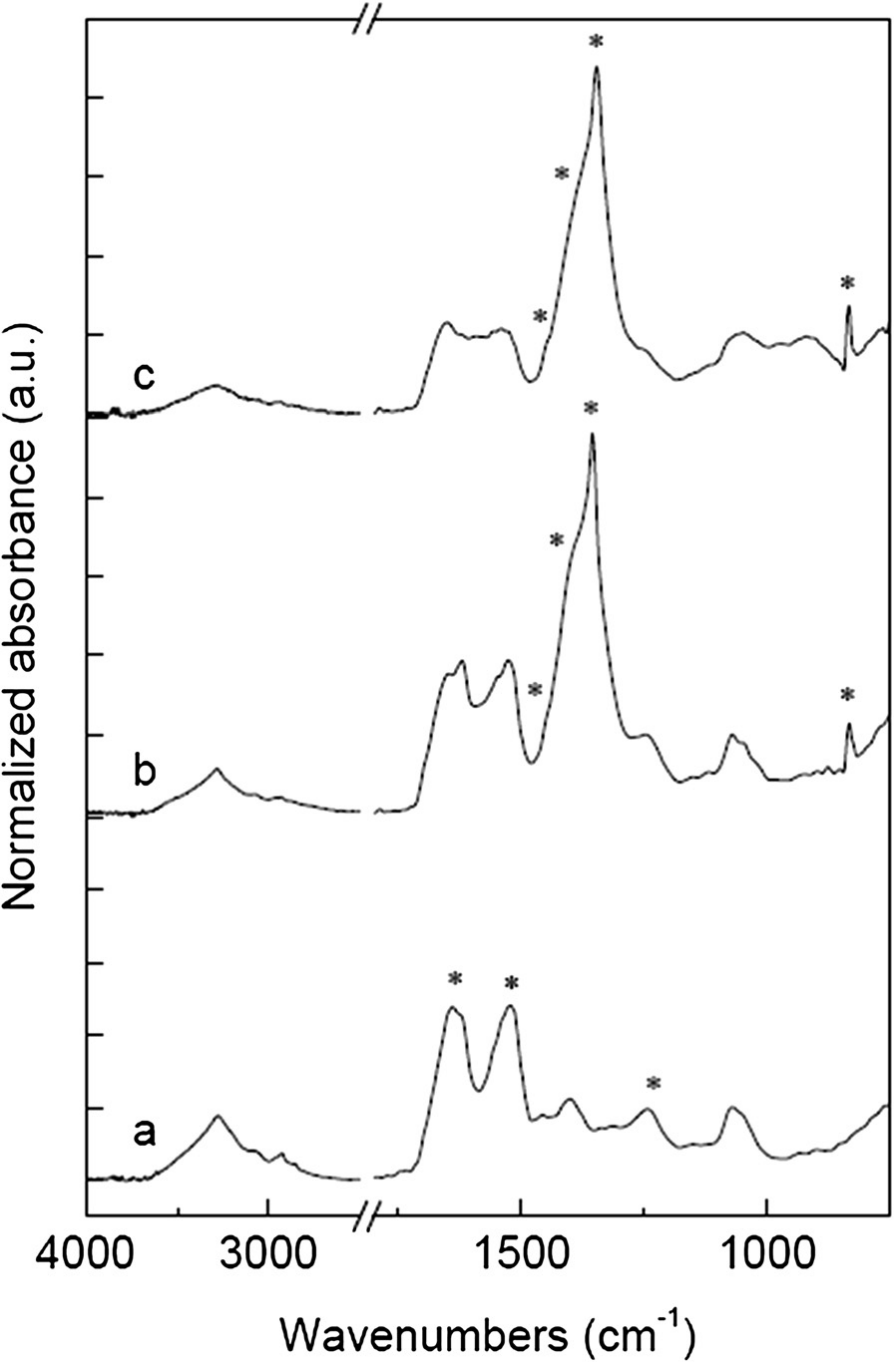
**Normalized ATR FT-IR spectra of virgin sericin and AgNPs.** The intensity was normalized against the absorption at 1,070 cm^−1^. **(A)** Original SS shows characteristic absorptions of protein including amide I (1,700 to 1,600 cm^−1^, asterisk), amide II (1,560 to 1,500 cm^−1^, asterisk), and amide III (1,300 to 1,200 cm^−1^, asterisk). **(B)** SS-capped AgNPs show new functional groups including carboxylate (1,451, 1,404, 1,353 cm^−1^, asterisk) and amine salt (830 cm^−1^, asterisk). **(C)** Thermally treated SS-capped AgNPs show the same functional groups as those in **(B)**.

Figure 4 and Table 4 show the normalized ATR FT-IR spectra and infrared band assignment of the original SS and SS-capped AgNPs. The spectrum of the original SS (Figure 4(A)) showed characteristic absorptions of protein including amide I (1,700 to 1,600 cm^−1^), amide II (1,560 to 1,500 cm^−1^), and amide III (1,300 to 1,200 cm^−1^) [33]. After the addition of silver salt under an alkaline condition (Figure 4(B)), an obvious presence of new functional groups including carboxylate (1,451, 1,404, 1,353 cm^−1^) and amine salt (830 cm^−1^) was observed. The new absorptions indicated the hydrolysis of amide linkage into its basic structural units [34]. The carboxylate groups also function as a weak reducing agent for the generation of AgNPs [35-38]. This FT-IR data proved the mechanism of how SS under an alkaline condition formed the AgNPs. In addition, the reduction reaction was accelerated by the thermal treatment of the SS-capped AgNPs (Figure 4(C)). The carboxylate (COO^−^) and amine (NH_2_ ^+^) moieties were clearly present. It was reported that these moieties could stabilize AgNPs through the donated lone pair electrons to the surface of metal nanoparticles [35,38]. An excellent stabilization of AgNPs by COO^−^ and NH_2_^+^ groups also prevented precipitation and aggregation of the nanoparticles [21,32].

**Table 4:** Infrared band assignment of silk sericin and SS-capped AgNPs

**IR band (cm**^**−1**^**)**	**Band assignment**
1,700 to 1,600	Amide I (C = O stretching vibration)
1,560 to 1,500	Amide II (N-H bending and C-N stretching vibration)
1,300 to 1,200	Amide III (C-N-H in-plane bending and C-N stretching vibration)
1,451, 1,404, 1,353	Free carboxylate groups (COO^−^ stretching vibration)
830	Amine salt

**Figure 5:**
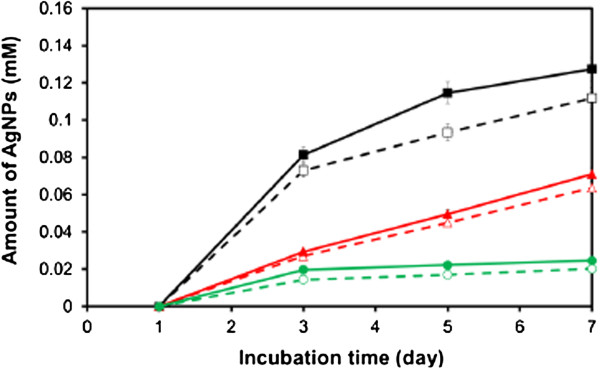
**Stability of SS-capped AgNPs synthesized at pH 11 when stored at different temperatures.** (empty square) 5 mg/mL SS + 5 mM AgNO_3_, 37°C, (filled square) 10 mg/mL SS + 5 mM AgNO_3_, 37°C, (empty triangle) 5 mg/mL SS + 5 mM AgNO_3_, 25°C, (filled triangle) 10 mg/mL SS + 5 mM AgNO_3_, 25°C, (empty circle) 5 mg/mL SS + 5 mM AgNO_3_, 4°C, (filled circle) 10 mg/mL SS + 5 mM AgNO_3_, 4°C.

The synthesized SS-capped AgNPs were stored at different temperatures (4°C, 25°C, and 37°C) for 7 days to observe their stability, as shown in Figure 5. It was found that the SS-capped AgNPs stored at 4°C were rather stable along the 7-day period; on the other hand, more SS-capped AgNPs were formed when stored at 25°C and 37°C. This might be that the reduction reaction continued at higher temperature.

The anti-bacterial activity of SS-capped AgNPs against gram-positive and gram-negative bacteria is shown in Table 5. The SS-capped AgNPs synthesized from 5 mg/mL SS + 5 mM AgNO_3_ were selected for the study due to their high yield and stability. We found that the growth of all gram-positive bacteria (MRSA, *S. aureus*, and *B. subtilis)* was potentially inhibited by SS-capped AgNPs (MIC 0.008 mM) while the SS-capped AgNPs inhibited the growth of gram-negative bacteria at a lower MIC (0.004 mM for *P. aeruginosa* and *E. coli* and 0.001 mM for *A. baumannii*)*.* This result clearly elucidated the effectiveness of our SS-capped AgNPs for anti-bacterial applications.

**Table 5:** **Anti-bacterial activity of SS-capped AgNPs (5 mg/mL SS + 5 mM AgNO**_**3**_**) against gram-positive and gram-negative bacteria**

**Gram**	**Bacterial strain**	**Concentration of SS-capped AgNPs (mM)**					**MIC (mM)**
		**0.001**	**0.004**	**0.008**	**0.017**	**0.034**	
+	MRSA	T	T/C	C	C	C	0.008
+	*S. aureus*	T	T/C	C	C	C	0.008
+	*B. subtilis*	T	T/C	C	C	C	0.008
−	*P. aeruginosa*	T	C	C	C	C	0.004
−	*A. baumannii*	C	C	C	C	C	0.001
−	*E. coli*	T	C	C	C	C	0.004

T, turbid suspension; T/C, slightly turbid suspension; C, clear solution.

## Conclusions

SS-capped AgNPs were successfully synthesized under an alkaline condition (pH 11) via a green chemistry approach using SS as a reducing and stabilizing agent. The higher concentrations of SS and AgNO_3_ increased the yield of SS-capped AgNPs. Sizes of the SS-capped AgNPs were around 48 to 117 nm. The FT-IR result proved that the carboxylate groups obtained from alkaline degradation of SS would be a reducing agent for the generation of AgNPs while COO^−^ and NH_2_^+^ groups stabilized the AgNPs and prevented their precipitation or aggregation. Furthermore, the SS-capped AgNPs showed potent anti-bacterial activity against various gram-positive and gram-negative bacteria. We therefore introduced the SS-capped AgNPs as a safe candidate for anti-bacterial applications.

## Competing interests

The authors declare that they have no competing interests.

## Authors’ contributions

PA designed all the experiments and interpreted and discussed all the results. NB did the microbiology study. JR drafted the manuscript. TT performed some parts of the experiments. SE ran the FT-IR experiment. All authors read and approved the final manuscript.
