# Correction: green synthesis of silk sericin-capped silver nanoparticles and their potent anti-bacterial activity

**DOI:** 10.1186/1556-276X-9-136

**Published:** 2014-03-24

**Authors:** Pornanong Aramwit, Nipaporn Bang, Juthamas Ratanavaraporn, Thanasorn Thanavibul, Sanong Ekgasit

**Affiliations:** 1 Bioactive Resources for Innovative Clinical Applications Research Unit and Department of Pharmacy Practice, Faculty of Pharmaceutical Sciences, Chulalongkorn University, 254 PhyaThai Road, Patumwan, Bangkok 10330, Thailand; 2 Department of Chemical Engineering, Faculty of Engineering, Chulalongkorn University, 254 PhyaThai Road, Patumwan, Bangkok 10330, Thailand; 3 Sensor Research Unit, Department of Chemistry, Faculty of Science, Chulalongkorn University, 254 PhyaThai Road, Patumwan, Bangkok 10330, Thailand

## Correction

The original version of this article [1] was published with an incomplete author list. The co-author Thanasorn Thanavibul has now been added to the author list.
